# Microplastic-Mediated Gene Expression Alterations and Cancer Risk: Insights from Toxicogenomic Analysis

**DOI:** 10.3390/ijms27188223

**Published:** 2026-09-15

**Authors:** Kyu-Shik Lee, Yeong Chae Kim, Hye-Ran Kim, Jongwan Kim

**Affiliations:** 1 Department of Pharmacology, College of Medicine, Dongguk University, Gyeongju 38066, Republic of Korea; there1@dongguk.ac.kr; 2 Department of Anatomy, College of Medicine, Dongguk University, Gyeongju 38066, Republic of Korea; rladudco0816@dumc.or.kr; 3 Department of Biomedical Laboratory Science, Dong-Eui Institute of Technology, Busan 47230, Republic of Korea

**Keywords:** cancer risk, carcinogenesis, micro- and nanoplastics, comparative toxicogenomics database

## Abstract

Micro- and nanoplastics (MNPs) are pervasive environmental contaminants that pose significant threats to ecosystems and the health of humans and other organisms. Increasing evidence indicates that MNP exposure can induce various biological disturbances, including cytotoxicity, chronic inflammation, endocrine disruption, oxidative stress, metabolic dysfunction, and cellular impairment. Many of these processes are closely associated with cancer initiation and progression. However, the molecular mechanisms underlying the relationship between MNP exposure and carcinogenesis remain unclear. This review summarizes the current evidence regarding MNP-associated alterations in gene expression and discusses their potential implications in cancer development and progression. We highlight the toxicogenomic insights derived from the Comparative Toxicogenomics Database (CTD), focusing on key microplastics, including polyethylene, polyethylene terephthalate, polystyrene, and polyvinyl chloride. Specifically, we discuss the chemical–gene interactions, disease associations, gene ontology annotations, pathway enrichment profiles, and chemical similarity networks linked to these polymers. Overall, the available toxicogenomic evidence implies that MNP exposure is associated with biological processes involved in oxidative stress responses, inflammatory signaling, immune dysregulation, metabolic alterations, and cell cycle control, all of which are implicated in carcinogenesis. Finally, we discuss the strengths and limitations of CTD-based toxicogenomic approaches and propose research directions to better understand the potential contribution of MNP exposure to cancer risk.

## 1. Introduction

Plastic materials are indispensable in modern society because of their versatility, durability, and cost-effectiveness, resulting in continuously increasing production and consumption worldwide [1,2,3,4]. However, their low biodegradability has led to their persistent accumulation in the environment, making plastic pollution one of the most pressing environmental challenges of recent decades [4,5,6,7]. Various strategies, including plastic recycling and waste management policies have been implemented to reduce the amount of plastic waste entering ecosystems [5,7,8,9,10]. However, these approaches have limited effectiveness in controlling microscopic plastic particles that are either manufactured for industrial and commercial applications or generated through the fragmentation and degradation of larger plastic products by physical, chemical, and biological processes [11,12]. Once released, these small particles are difficult to recover and contribute substantially to environmental contamination [13,14,15].

**Figure 1:**
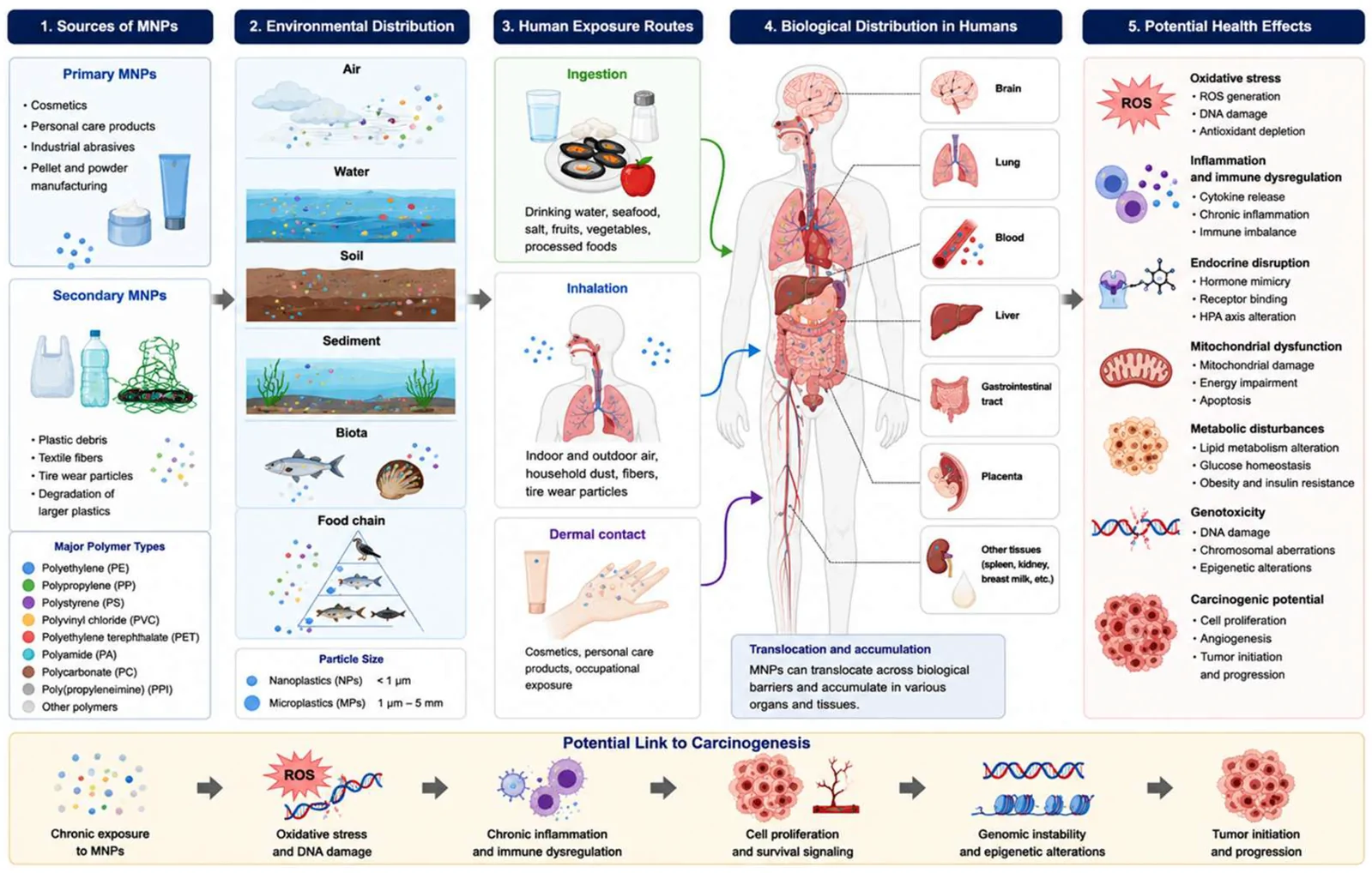
Environmental sources, human exposure pathways, biological distribution, and potential health effects of MNPs. This schematic illustrates the overall lifecycle of human exposure to MNPs, from their environmental origins to their potential biological consequences. MNPs originate from both primary sources, including cosmetics, personal care products, industrial abrasives, and manufactured plastic particles, and secondary sources generated through the environmental degradation and fragmentation of larger plastic products, textile fibers, and tire wear particles. These particles become widely distributed across environmental compartments, including air, water, soil, sediments, and aquatic food chains. Humans are continuously exposed to MNPs through multiple pathways, including dietary ingestion, inhalation of airborne particles, and, to a lesser extent, dermal contact. Following exposure, MNPs may translocate across biological barriers and distribute to multiple organs and tissues, including the gastrointestinal tract, lungs, liver, blood, placenta, brain, and other peripheral tissues. Although the extent of tissue accumulation and long-term retention remains under investigation, increasing evidence suggests that MNPs can reach systemic circulation and interact with diverse biological systems. Experimental studies have associated MNP exposure with multiple adverse biological responses, including oxidative stress, chronic inflammation and immune dysregulation, endocrine disruption, mitochondrial dysfunction, metabolic disturbances, and genotoxicity. These interrelated molecular and cellular alterations may cooperatively contribute to processes relevant to carcinogenesis. As summarized in the lower panel, chronic MNP exposure may promote a sequence of biological events involving oxidative stress and DNA damage, persistent inflammatory responses, dysregulated cell proliferation and survival signaling, genomic instability, and epigenetic alterations, thereby creating biological conditions that could facilitate tumor initiation and progression. However, these proposed mechanisms should be interpreted as biologically plausible pathways supported by current experimental evidence rather than definitive evidence of carcinogenicity.

Microplastics (MPs) are commonly defined as plastic particles smaller than 5 mm in diameter, whereas nanoplastics (NPs) are typically considered particles measuring less than 1 μm [16,17,18,19]. Broadly referred to as micro- and nanoplastics (MNPs), these contaminants are characterized by their insolubility in water, diverse morphologies, heterogeneous polymer compositions, and remarkable environmental persistence [20,21,22,23]. Because of these properties, MNPs have been detected in marine and freshwater ecosystems, soils, and the atmosphere [24,25,26]. Through environmental circulation and food webs, MNPs can accumulate in a wide variety of organisms, raising concerns about their potential impact on ecosystem integrity and human health (Figure 1) [3,27,28,29].

Humans are exposed to MNPs primarily through ingestion of contaminated food and drinking water, inhalation of airborne particles, and, to a lesser extent, dermal contact [30,31]. MNPs have been detected in human biological samples, including the placenta, meconium, breast milk, blood, and feces [32,33,34,35,36]. Accumulating evidence suggests that MNP accumulation in the human body may be associated with physiological dysfunction, including immune dysregulation, impaired reproductive capacity, and developmental abnormalities [37,38]. Furthermore, MNP exposure has been linked to chronic inflammation, metabolic disorders, cardiovascular diseases, and other adverse health outcomes [38,39]. These findings have raised concerns about the potential contribution of chronic MNP exposure to the development of complex human diseases, including cancer [38].

At the molecular and cellular levels, MNPs induce a broad spectrum of biological responses. A consistently reported mechanism underlying MNP toxicity is the induction of oxidative stress through excessive production of reactive oxygen species (ROS), which results in oxidative damage to DNA, proteins, and lipids [3,16,17]. In addition, MNPs can activate inflammatory signaling pathways, including NF-κB, MAPK, and inflammasome-linked pathways, leading to altered cytokine production and chronic inflammatory responses [3,38]. MNP exposure has also been associated with mitochondrial dysfunction, endoplasmic reticulum stress, autophagy, apoptosis, endocrine disruption, metabolic disturbances, and epigenetic modifications [16,17,28]. Moreover, MNPs are likely to function as carriers of other environmental pollutants, including heavy metals and persistent organic pollutants, thereby amplifying their toxicological effects [12,15]. Taken together, the available evidence indicates that MNPs may affect interrelated molecular pathways rather than isolated biological processes.

Notably, many of these alterations overlap with established mechanisms of carcinogenesis [38,40]. Oxidative stress promotes DNA damage and genomic instability, chronic inflammation contributes to tumor-promoting microenvironments, immune dysregulation facilitates immune evasion, and metabolic disturbances support the energy demands of proliferating cancer cells [40,41]. Furthermore, the disruption of apoptosis and deregulation of cell cycle progression may enable the survival and expansion of transformed cells [40]. Experimental studies have demonstrated that MNP exposure can modulate oncogenic and tumor suppressor signaling pathways, alter inflammatory responses, and affect cellular processes involved in tumor initiation and progression [42,43,44]. These findings suggest that the potential contribution of MNP exposure to carcinogenesis may involve the coordinated perturbation of multiple intertwined biological networks, rather than from a single molecular event (Figure 1). However, whether these molecular responses converge on common mechanisms shared by different MNP polymers remains largely unknown.

Toxicogenomics has improved our understanding of environmentally induced diseases by integrating chemical exposure data with gene expression profiles, biological pathways, and disease associations [45]. Unlike conventional toxicological approaches that primarily evaluate phenotypic outcomes, toxicogenomic analyses enable the systematic exploration of the molecular networks underlying chemical toxicity and facilitate the generation of mechanistic hypotheses relevant to human diseases [45,46]. The Comparative Toxicogenomics Database (CTD; https://ctdbase.org/; accessed on 15 June 2026) is a comprehensive manually curated database that integrates experimentally supported chemical–gene interactions, chemical–disease associations, gene–disease relationships, Gene Ontology (GO) annotations, biological pathways, phenotypes, and exposure information [47], thus providing a valuable system-level resource for identifying molecular pathways and biological processes potentially linked to environmentally induced diseases [47,48].

Although individual MNP polymers can alter gene expression and perturb diverse biological networks, these responses have been largely investigated independently across polymers and experimental models [16,43,44,49]. Therefore, the underlying carcinogenesis-linked mechanisms remain unclear. Systematic comparisons of key MNP polymers are limited, hindering a comprehensive understanding of shared and polymer-specific biological effects. To address this knowledge gap, the present review integrates CTD-derived toxicogenomic information for four selected environmental MP polymers: polyethylene (PE), polyethylene terephthalate (PET), polystyrene (PS), and polyvinyl chloride (PVC) [48,50]. Systematic analyses of chemical–gene interactions, disease associations, GO annotations, pathway enrichment profiles, and chemical similarity networks were conducted to identify both polymer-specific molecular characteristics and common molecular signatures associated with MNP exposure [47,48,50]. Polypropylene (PP), despite being one of the most abundant environmental plastics, was excluded from the comparative analysis because of the limited availability of PP-specific toxicogenomic information in the current CTD database, precluding meaningful comparative interpretation.

Rather than establishing causality, this review integrates existing molecular evidence to define the biological processes putatively linked to MNP exposure and carcinogenesis. By highlighting convergent toxicogenomic signatures, this review proposes that oxidative stress, chronic inflammation, dysregulated cell survival, and metabolic reprogramming may act as integrated molecular processes, rather than independent toxicological events. This model may help prioritize candidate targets and pathways for future mechanistic and epidemiological investigations [45,46].

## 2. Overview of MNPs and Human Exposure

MNPs are a highly heterogeneous group of plastic particles that differ substantially in their physicochemical properties, environmental behavior, and biological interactions. Although MNPs are commonly classified according to their particle size, their environmental fate and biological effects are influenced by several factors, including polymer composition, particle morphology, surface chemistry, weathering processes, and associated chemical additives [16,21,22]. These characteristics determine the persistence and transport of MNPs in the environment and influence their routes of human exposure, biodistribution, and interactions with biological systems [21,22,28,51]. Therefore, understanding the physicochemical diversity of MNPs and their exposure characteristics is fundamental for interpreting toxicological observations and evaluating their potential implications for human health [52]. The following sections summarize the major physicochemical characteristics of representative MNPs, the principal routes of human exposure, current evidence regarding their distribution in human tissues, and the methodological challenges inherent in assessing human exposure.

### 2.1. Major Types and Physicochemical Characteristics of MNPs

Of the wide variety of synthetic polymers produced globally, PE, PP, PET, PS, PVC, PA, and PC constitute the dominant sources of environmental MNP contamination because of their extensive applications in packaging materials, food containers, textiles, medical devices, construction materials, and consumer products [1,10,21,22]. Environmental monitoring studies have consistently identified these polymers as the most abundant plastic particles in aquatic ecosystems, soil, atmospheric environments, and food products [21,24,25].

Particle size is a primary factor governing the environmental behavior and biological fate of MNPs. Compared with MPs, NPs possess substantially larger specific surface areas and higher surface reactivities, enabling stronger interactions with dissolved organic matter, biomolecules, and cellular membranes [19,20,51]. Their small dimensions further facilitate the penetration of biological barriers and increase the probability of intracellular localization following exposure. Consequently, particle size influences not only environmental mobility, but also biodistribution and biological accessibility. These size-dependent differences in biological accessibility may also influence the magnitude and nature of the toxicological responses induced by MPs and NPs. Although both particle classes can induce common responses, including oxidative stress, inflammation, and apoptosis, NPs generally show greater cellular uptake and intracellular localization than larger MPs and may therefore exert more pronounced effects on intracellular signaling pathways [53,54]. Experimental studies have suggested that NP exposure can modulate signaling pathways involved in cell proliferation, survival, migration, and invasion, including those relevant to tumor progression [55,56,57]. In contrast, larger MPs are less readily internalized at the cellular level and may instead exert substantial effects at sites of direct exposure, particularly in the gastrointestinal tract, where persistent particle accumulation may contribute to mucosal injury, oxidative stress, and chronic inflammation [58,59]. Thus, MPs and NPs may affect cancer-related biological processes through partially distinct pathways [54,60]. Nevertheless, these observations primarily derive from experimental models and indicate biological plausibility rather than a demonstrated causal relationship between MP or NP exposure and human cancer [54,60].

Particle morphology is another important characteristic affecting MNP behavior. Environmental MNPs occur in diverse forms, including fragments, fibers, films, foams, pellets, and spherical particles, depending on their original applications and degradation processes [12,22]. Fibrous MPs released from synthetic textiles are particularly prevalent in both indoor and outdoor environments and exhibit distinct aerodynamic properties compared to irregular fragments, resulting in differences in transport, deposition, and persistence following inhalation exposure [30,61,62]. Similarly, the surface roughness and particle geometry influence the adsorption capacity, aggregation behavior, and interactions with the surrounding biological matrices.

The physicochemical properties of MNPs are affected by environmental weathering. Exposure to ultraviolet radiation, thermal cycling, oxidation, mechanical abrasion, and microbial degradation alter particle morphology, surface chemistry, crystallinity, and mechanical stability [63,64,65]. Weathered particles typically exhibit increased surface roughness, oxygen-containing functional groups, and enhanced adsorption capacities relative to those of pristine plastics, resulting in substantially different environmental behaviors. As most environmental MNPs undergo varying degrees of aging before human exposure, weathered particles are increasingly being recognized as environmentally relevant models for toxicological investigations. Another important characteristic of environmental MNPs is their ability to interact with exogenous chemicals. In addition to intentionally incorporated additives, such as plasticizers, antioxidants, pigments, flame retardants, and ultraviolet stabilizers, weathered plastic particles readily adsorb environmental contaminants, including heavy metals, polycyclic aromatic hydrocarbons, persistent organic pollutants, pharmaceutical residues, and microorganisms [66,67]. These interactions alter the physicochemical properties of MNPs and influence their environmental transport, bioavailability, and biological properties. Consequently, the potential health effects of MNP exposure should be considered within the broader context of complex environmental mixtures rather than solely attributable to the polymer matrix itself. Overall, the diversity of polymer compositions and physicochemical characteristics highlights the complexity of environmental MNPs. These characteristics influence not only the environmental fate of plastic particles but also the extent and nature of human exposure. Therefore, the consideration of particle heterogeneity is essential for interpreting experimental findings and comparing toxicological responses between different MNP polymers, particularly in comparative toxicogenomic analyses.

### 2.2. Human Exposure Pathways to MNPs

Human exposure to MNPs occurs continuously through a wide range of environmental and occupational sources. The principal exposure routes include dietary ingestion, inhalation of airborne particles, and dermal contact, although the relative contribution of each pathway remains difficult to quantify due to differences in analytical methodologies and environmental conditions [30,31,51].

Dietary ingestion is generally considered the predominant route of MNP exposure. Plastic particles have been detected in a wide variety of food products, including seafood, drinking water, bottled water, table salt, honey, beer, fruits, vegetables, and processed foods [68,69,70,71]. Marine organisms, particularly filter feeding species, such as mussels and oysters, readily accumulate MNPs from contaminated aquatic environments, facilitating trophic transfer through food webs [24,51]. Food processing, packaging, storage, and preparation can also introduce secondary plastic contamination, which further increases the likelihood of dietary exposure.

Inhalation is another important exposure pathway, particularly in urban and indoor environments, where airborne plastic particles are abundant. Synthetic textile fibers released from clothing, carpets, upholstered furniture, household dust, and other environmental sources are major contributors to indoor airborne MNPs, whereas tire wear particles and road dust are important outdoor sources [30,61,62]. Owing to their small aerodynamic diameters, fine MNPs may remain suspended in the air for extended periods, thereby increasing the probability of inhalation exposure.

Dermal contact is widely regarded as a comparatively minor route of systemic MNP exposure because the intact human skin provides an effective physical barrier against most plastic particles [30,72]. However, localized exposure may occur through contact with contaminated materials or consumer products, while the extent to which smaller particles can penetrate compromised skin remains uncertain.

Human exposure rarely occurs through a single route. Instead, individuals are continuously exposed to complex mixtures of MNPs originating from food, drinking water, air, consumer products, and occupational environments [30,31]. Such exposure complexity should be considered when evaluating the biological effects of MNPs and comparing the toxicological findings obtained from different experimental studies.

### 2.3. Internal Distribution and Bioaccumulation of MNPs in Humans

Increasing evidence indicates that MNPs can translocate beyond their initial sites of exposure and are distributed to diverse organs and tissues throughout the human body [49,51,52]. The detection of MNPs in human blood provides direct evidence that plastic particles can enter the systemic circulation following gastrointestinal absorption or pulmonary uptake [35,51,52]. Once in the bloodstream, MNPs may interact with plasma proteins, immune cells, and vascular endothelial barriers, thereby facilitating their transport to distant organs [51,72,73]. Although the efficiency of systemic translocation remains incompletely understood, available evidence suggests that particle size, surface characteristics, and protein corona formation are important determinants of biodistribution [49,51].

MNPs have also been detected in human placental tissues, meconium and breast milk, suggesting maternal-fetal transfer and exposure during early developmental stages [32,33,34,74,75]. In addition, their detection in surgically resected human lung tissues supports the deposition of inhaled particles within the respiratory tissues, while their presence in feces reflects continuous dietary exposure and gastrointestinal elimination [36,62,76].

Collectively, these findings indicate that MNPs can reach various biological compartments following exposure, although the mechanisms governing their absorption, distribution, metabolism, and excretion remain unclear. Current evidence suggests that biodistribution of MNPs is influenced by several physicochemical characteristics, including particle size, morphology, surface chemistry, polymer composition, and host-specific physiological factors [49,51,72]. However, the long-term fate of the retained particles and the biological consequences of chronic tissue accumulation remain largely unknown.

### 2.4. Challenges in Assessing Human Exposure to MNPs

Despite the rapidly increasing interest in the potential health effects of MNP exposure, the accurate assessment of human exposure remains a considerable scientific challenge [49,52]. One of the primary limitations is the lack of standardized methodologies for sampling, isolation, identification, and quantification of MNPs in different environmental and biological matrices. Analytical techniques, including Fourier-transform infrared spectroscopy, Raman spectroscopy, pyrolysis–gas chromatography/mass spectrometry, and electron microscopy, possess distinct strengths and limitations, resulting in substantial interstudy variability [22,29].

The detection and characterization of NPs present additional technical challenges owing to their extremely small particle sizes, low environmental concentrations, and complex interactions with biological matrices [19,29]. Consequently, the current estimates of human exposure are likely to underestimate the true burden of environmental MNP contamination.

Another important limitation is the considerable heterogeneity among experimental studies. Differences in polymer composition, particle size, morphology, weathering status, exposure concentrations, exposure duration, biological models, and analytical methodologies complicate direct comparisons of independent investigations and hinder the establishment of reliable exposure thresholds relevant to human health risk assessment [16,22,29]. These methodological inconsistencies also impede the identification of molecular responses consistently elicited by different MNP polymers.

To address these challenges, future studies should prioritize the development of standardized analytical protocols, harmonized reporting guidelines, environmentally relevant exposure models, and integrated data analysis strategies to improve the reliability and comparability of toxicological findings [29,47]. Such efforts will substantially strengthen human health risk assessments and facilitate the identification of robust biomarkers of MNP exposure and toxicity.

Collectively, the diversity of MNPs, complexity of human exposure, and heterogeneity of existing experimental evidence highlight the need for integrative approaches capable of systematically organizing molecular information generated from different polymer types. Toxicogenomic resources provide valuable opportunities to compare molecular responses, identify common biological pathways, and generate mechanistic hypotheses regarding the potential health effects of MNP exposure. The following section therefore introduces CTD as a comprehensive platform for comparative toxicogenomic analyses of selected environmental MNP polymers [45,46,47].

## 3. CTD-Based Toxicogenomic Approach

### 3.1. Toxicogenomics in Environmental Health Research

Understanding the molecular mechanisms by which environmental contaminants contribute to human diseases remains a major challenge in environmental toxicology [45,46]. Conventional toxicological studies have primarily focused on phenotypic endpoints, including cytotoxicity, oxidative stress, histopathological alterations, and organ-specific toxicity [45,46]. Although these approaches provide valuable information regarding adverse biological outcomes, they often provide limited insight into the complex molecular networks underlying toxicant-induced diseases.

Recent advances in high-throughput technologies have transformed environmental health research from descriptive toxicology to systems toxicology [45,46,77]. Toxicogenomics integrates transcriptomics, genomics, proteomics, metabolomics, and computational biology to comprehensively investigate the molecular responses following chemical exposure. Rather than evaluating individual genes or signaling pathways independently, toxicogenomic approaches enable the simultaneous investigation of complex gene networks, biological pathways, and disease associations, thereby facilitating a system-level understanding of chemical toxicity. Importantly, toxicogenomics provides opportunities not only to identify molecular biomarkers of exposure but also to generate mechanistic hypotheses linking environmental contaminants to human diseases [45,78].

These approaches have become increasingly valuable for investigating chronic diseases with multifactorial etiologies, including cancer, cardiovascular diseases, metabolic disorders, and neurodegenerative diseases. Among the wide range of environmental contaminants currently under investigation, MNPs are a particularly challenging research target because their biological effects are highly heterogeneous and depend on multiple physicochemical and exposure-related variables, making cross-study comparisons difficult [16,43,49,51].

### 3.2. Overview of CTD

CTD is a publicly available, manually curated database developed to facilitate investigations into the relationships between environmental chemicals, genes, phenotypes, biological pathways, and human diseases. Unlike conventional toxicological databases, which primarily provide chemical properties or toxicity classifications, CTD focuses on molecular interactions supported by published experimental evidence [47,48].

Currently, CTD contains millions of manually curated and inferred relationships involving chemicals, genes, diseases, GO annotations, biological pathways, phenotypes, and exposure information. Data were extracted from peer-reviewed publications by expert biocurators and standardized using controlled vocabularies, such as Medical Subject Headings, GO, KEGG, WikiPathways, Reactome, and Disease Ontology [47]. Consequently, CTD is one of the most comprehensive resources for environmental toxicogenomic research.

A major advantage of CTD is its ability to integrate diverse molecular relationships into interlinked biological networks. For a given chemical, CTD provides experimentally supported chemical–gene interactions, inferred chemical–disease associations, enriched GO terms, biological pathways, phenotype annotations, and chemical similarity networks [47,48]. These integrated datasets facilitate the identification of molecular signatures that may not be readily apparent in individual experimental studies.

Although CTD has been extensively used to investigate the molecular toxicity of diverse environmental chemicals, direct comparative toxicogenomic analyses of the major environmentally relevant MP polymers remain largely unexplored [43,47]. Therefore, this review applies a unified CTD-based analytical approach to systematically compare the toxicogenomic profiles of PE, PET, PS, and PVC, thereby identifying both the polymer-specific characteristics and convergent molecular patterns potentially implicated in carcinogenesis.

### 3.3. CTD-Derived Toxicogenomic Information Used in This Review

To comprehensively evaluate the molecular processes putatively linking MNP exposure to carcinogenesis, this review integrates several complementary toxicogenomic datasets available in CTD. Rather than relying on a single category of molecular information, these datasets were interpreted collectively to provide a system-level understanding of the biological responses associated with different MP polymers.

#### 3.3.1. Chemical–Gene Interactions

Chemical–gene interaction data constitute the foundation of CTD-based toxicogenomic analyses. These curated datasets summarize the experimentally validated interactions between individual chemicals and specific genes, including alterations in gene expression, protein abundance, enzyme activity, molecular binding, and post-translational modifications. Analysis of these interactions enables the identification of genes that are consistently responsive to MNP exposure and provides a basis for subsequent functional interpretation.

#### 3.3.2. Gene-Disease Associations

CTD integrates chemical–gene interactions with curated gene–disease relationships to infer potential disease associations. Although these inferred associations do not establish direct causality, they identify diseases that share common molecular signatures with chemical-responsive genes, thereby providing valuable hypotheses regarding the potential health outcomes resulting from environmental exposure.

#### 3.3.3. GO Enrichment Analysis

GO enrichment analysis facilitates functional interpretation of chemical-responsive genes by classifying them according to their biological processes, molecular functions, and cellular components. GO enrichment enables the identification of biological processes that are preferentially enriched in chemical-responsive genes, thus providing functional insights beyond individual gene-level observations.

#### 3.3.4. Pathway Enrichment Analysis

Pathway analyses integrate chemical-responsive genes into established signaling networks, including the KEGG, Reactome, and WikiPathways. The identification of enriched pathways allows researchers to evaluate whether numerous responsive genes converge on common biological mechanisms, including oxidative stress responses, inflammatory signaling, cell cycle regulation, apoptosis, DNA repair, and cancer-related signaling pathways.

#### 3.3.5. Phenotype Annotations

CTD phenotype annotations provide curated information on biological processes and cellular responses affected by chemical exposure. These data describe changes in diverse phenotypes, including cell proliferation, oxidative stress responses, inflammatory processes, mitochondrial function, and metabolic regulation. Analysis of phenotype annotations complements chemical–gene and pathway information by providing additional context for the biological responses associated with individual MNP polymers. Co-mentioned chemicals were additionally examined, and records explicitly describing co-treatment or combined exposure were distinguished from other co-mentioned relationships.

#### 3.3.6. Chemical Similarity Networks

Unlike pure structural similarity analyses, CTD chemical similarity networks integrate common chemical–gene interaction profiles with structural attributes. Chemicals exhibiting similar interaction patterns could influence overlapping biological pathways despite possessing distinct chemical structures. Consequently, chemical similarity analyses facilitate the prediction of shared molecular mechanisms and provide additional evidence to support toxicity-related hypotheses.

### 3.4. Advantages and Limitations of CTD-Based Toxicogenomic Analyses

The integration of CTD-derived toxicogenomic information offers several important advantages in environmental health research [47,48]. First, CTD enables systematic comparison of different environmental contaminants using a unified analytical strategy. Second, the integration of chemical–gene interactions with disease associations, GO annotations, and biological pathways provides a systems-level understanding of chemical toxicity. Third, the manually curated nature of CTD substantially improves data reliability compared to automated text-mining databases.

Nevertheless, several limitations should be considered when interpreting CTD-derived information [47]. Most importantly, CTD identifies associations rather than direct causal relationships. Chemical–gene interactions originate from heterogeneous experimental systems involving different exposure concentrations, exposure durations, biological models, and analytical methodologies. Consequently, the inferred disease associations should be interpreted as hypothesis-generating evidence that requires subsequent experimental validation. Furthermore, publication bias can influence the availability of curated interactions, particularly for the relatively understudied environmental contaminants.

Despite these limitations, CTD provides a robust platform for systematically integrating the heterogeneous molecular evidence generated from independent experimental studies. Rather than establishing causal relationships, CTD facilitates the comparative identification of conserved molecular responses, thereby enabling the generation of biologically plausible hypotheses that can be subsequently validated experimentally. In the present review, a comparative toxicogenomic approach was applied to identify polymer-specific toxicogenomic characteristics and conserved molecular mechanisms that might putatively link environmentally relevant MNP polymers to carcinogenesis.

## 4. Comparative Toxicogenomic Profiles of Major MNPs

To characterize the molecular responses implicated in the relationship between MNP exposure and carcinogenesis, CTD-derived toxicogenomic profiles were comparatively reviewed for PE, PET, PS, and PVC. The analysis focused on chemical–gene interactions, disease associations, GO enrichment, pathway enrichment, phenotype annotations, and chemical similarity profiles [47]. Although each polymer exhibited a distinct toxicogenomic profile, biological processes related to oxidative stress, inflammatory signaling, immune regulation, apoptosis, metabolic regulation, angiogenesis, and cell cycle regulation were repeatedly enriched in the CTD-derived datasets.

### 4.1. PE

PE is the most widely produced plastic polymer and one of the predominant constituents of environmental MPs [1,10]. Owing to its widespread occurrence, considerable attention has been directed toward understanding the molecular responses to PE exposure. CTD-derived toxicogenomic analysis revealed that PE-responsive genes were frequently involved in biological processes related to inflammation, oxidative stress, apoptosis, and cell survival (Table 1 and Data S1). Among these genes, *IL6*, *TNF*, *CAT*, *HMOX1*, *KEAP1*, and *NFE2L2* were prominently represented, with inflammatory signaling and redox regulation as major features of the PE-associated interaction profile. Other genes, including *IL1B*, *AKT1*, *BAX*, and *BCL2*, connected the PE data set to inflammatory and cell survival-related processes (Table 1). The results of functional enrichment analyses were consistent with these observations (Data S1). GO enrichment identified terms related to the negative regulation of the apoptotic process, negative regulation of programmed cell death, and system development, whereas pathway enrichment analyses showed enrichment of cytokine signaling, interleukin signaling, immune system pathways, and endocrine resistance. Taken together, these results suggest that inflammatory signaling, redox homeostasis, and cell survival constitute major components of the PE-associated toxicogenomic profile.

CTD-derived disease inference analysis identified potential associations between PE-responsive genes and several neoplastic conditions, including breast neoplasms, prostatic neoplasms, urinary bladder neoplasms, neoplasm invasiveness, and neoplastic cell transformation (Table 1). Chemical similarity analysis revealed compounds sharing toxicogenomic interaction patterns involving oxidative stress, inflammatory signaling, and epithelial function (Data S1).

### 4.2. PET

PET is one of the most extensively manufactured polyester polymers and is widely used in beverage bottles, food packaging, synthetic fibers, and medical materials [1,10]. The continuous environmental release and fragmentation of PET products have contributed to the widespread detection of PET-derived MNPs in aquatic environments, terrestrial ecosystems, and food chains [79,80]. Although PET is generally regarded as chemically stable, numerous experimental studies have indicated that exposure to PET-derived MPs induces inflammatory responses, oxidative stress, and metabolic alterations under certain experimental conditions [81,82]. CTD-derived toxicogenomic analysis revealed that PET may exhibit a molecular profile differed from those of the other selected MNP polymers (Table 1 and Data S2). PET-responsive genes included *IL1B*, *IL6*, *TNF*, *APOA4*, *AGPAT1*, *GPAT4*, *LPCAT3*, *MOGAT3*, *MGLL*, *MFSD2A*, *HMGCS2*, and *PLCE1*, linking the PET dataset to inflammatory signaling and lipid metabolism regulation (Table 1). Compared with PE, PET-responsive genes showed greater enrichment in glycerolipid metabolism, phospholipid remodeling, and lipid homeostasis, implying that lipid metabolic processes might constitute a relatively prominent feature of the PET toxicogenomic profile (Datas S1 and S2).

Functional enrichment analyses were reinforced this pattern. GO enrichment identified biological processes, including glycerolipid metabolism, cellular lipid metabolism, lipid metabolic processes, and response to stimuli, whereas molecular function analysis highlighted cytokine activity, protein binding, and transferase activity (Data S2). Similarly, pathway enrichment analyses revealed metabolic pathways, lipid and lipoprotein metabolism, phosphatidylinositol signaling, and inositol phosphate metabolism as the most highly enriched pathways (Data S2). Taken together, these findings show that lipid metabolic regulation is a prominent component of the PET-associated toxicogenomic profile.

CTD-derived disease inference analysis identified potential links between PET-responsive genes and several neoplastic conditions, including breast, lung, stomach, and hepatocellular carcinomas (Table 1). Phenotype annotations also included cytokine production, apoptotic processes, cell migration, reactive oxygen species metabolism, and intestinal barrier-related processes (Data S2), consistent with previous experimental studies describing inflammatory and cellular responses following PET-derived MNP exposure [83,84,85]. In addition, chemical similarity analysis identified several compounds sharing comparable toxicogenomic interaction profiles, particularly involving genes related to lipid metabolism and inflammatory signaling (Data S2).

### 4.3. PS

PS is one of the most extensively studied MP polymers owing to its widespread use in food packaging, disposable containers, laboratory materials, and consumer products [10,86]. Owing to its environmental persistence and propensity to fragment into micro- and nano-sized particles, PS has frequently been used as a model polymer in experimental toxicology. A comparative evaluation of CTD-derived toxicogenomic datasets showed that PS had a relatively extensive molecular interaction dataset compared to the other selected MNP polymers examined in this review. This difference may reflect the greater availability of PS-related experimental data in CTD and should not be interpreted as evidence of greater biological toxicity (Table 1 and Datas S1—S4).

The CTD-derived gene interaction profile included numerous genes involved in inflammation, oxidative stress, apoptosis, immune regulation, and cancer-related signaling. These genes included *TNF*, *APOE*, *IL6*, *IL1B*, *BCL2*, *CASP8*, *TP53*, *AKT1*, *MYC*, *STAT3*, *PTEN*, *HMOX1*, *CAT*, *NFKB1*, *RELA*, *PTGS2*, *TGFB1*, *MMP2*, and *MMP9*, linking to these biological processes (Table 1).

Functional enrichment showed a similarly broad pattern. GO enrichment analysis identified categories related to cellular processes, responses to stimuli, metabolic processes, positive regulation of biological processes, and biological regulation (Data S3). Rather than being concentrated within a single dominant biological process, PS-responsive genes were distributed across diverse cellular stress, metabolic, and regulatory processes. Pathway enrichment analysis further identified pathways in cancer, immune system, signal transduction, signaling by interleukins, innate immune system, and TNF signaling pathway. Pathways in the cancer category included *AKT1*, *BAX*, *BCL2*, *CASP3*, *CASP8*, *CCND1*, *CDKN1A*, *CTNNB1*, *HIF1A*, *IL6*, *MTOR*, *MYC*, *PTEN*, *RELA*, *STAT3*, *TGFB1*, and *TP53* (Table 1), while the enrichment of the TNF and interleukin signaling pathways were also enriched in the PS toxicogenomic profile.

The CTD-derived disease inference analysis revealed associations between PS-responsive genes and several human disease categories, including obesity, hypertension, non-alcoholic fatty liver disease, inflammation, chemical- and drug-induced liver injury, and kidney disease (Table 1). Phenotype annotation covered ROS metabolism, autophagy, cell death, immune activation, and inflammatory responses, broadly consistent with previous in vitro and in vivo studies describing oxidative stress, mitochondrial dysfunction, inflammasome activation, and apoptosis following PS MP exposure [58,87,88,89,90]. Chemical similarity analysis identified compounds sharing comparable toxicogenomic interaction profiles, particularly with respect to oxidative stress and inflammatory signaling (Data S3).

### 4.4. PVC

PVC is one of the most widely produced synthetic polymers and is used extensively in construction materials, medical devices, electrical insulation, and food packaging [10]. The environmental degradation of PVC products generates MNPs that are increasingly detected in terrestrial and aquatic environments [91,92]. Comparative analysis of CTD-derived toxicogenomic datasets suggested that PVC exhibits a more focused toxicogenomic profile than the other major MNP polymers examined in this review, with genes predominantly involved in oxidative stress- and inflammation-related pathways (Table 1 and Data S4). The CTD-derived gene interaction profile included *CAT*, *HMOX1*, *KEAP1*, *NFE2L2*, *IL1B*, *IL6*, *TNF*, *RELA*, and *ANGPT1*, which were among the genes most frequently associated with PVC (Table 1). The involvement of *NFE2L2 (NRF2)*, *KEAP1*, *HMOX1*, and *CAT* emphasizes redox homeostasis and antioxidant defense within PVC-associated dataset, together with inflammatory regulation involving *IL1B*, *IL6*, *TNF*, and *RELA*.

Functional enrichment analyses were consistent with these observations. GO enrichment identified biological processes related to the negative regulation of apoptosis and cell death, tube development, angiogenesis, and responses to hydrogen peroxide (Data S4). Pathway enrichment analyses included interleukins, immune system, IL-17 signaling pathway, fluid shear stress, atherosclerosis, and inflammatory bowel disease (Data S4). These results link the relatively focused PVC-associated gene set to redox regulation, inflammatory signaling, cell survival, and vascular processes.

CTD-derived disease inference analysis revealed potential associations with lung neoplasms, hepatocellular carcinoma, liver neoplasms, and general neoplasms, involving genes such as *HMOX1*, *IL1B*, *IL6*, *NFE2L2*, *TNF*, and *CAT* (Table 1). Phenotype annotations included inflammatory responses, leukocyte migration, necrotic cell death, and tissue remodeling (Data S4), in agreement with experimental evidence of inflammatory responses and oxidative stress following PVC-associated MNPs [93,94,95]. Chemical similarity analysis also identified compounds with partially overlapping interaction patterns, particularly with respect to oxidative stress regulation and inflammatory signaling (Data S4).

### 4.5. Comparative Toxicogenomic Profiles of Selected MNPs

A comparative evaluation of the CTD-derived toxicogenomic datasets showed that the selected MNPs shared both common and polymer-specific molecular characteristics. Although each polymer exhibited a distinct toxicogenomic profile, a comparison of the chemical–gene interactions, functional enrichment analyses, disease inference networks, chemical similarity profiles, and pathway annotations revealed several recurring biological themes within the analyzed datasets (Table 1). These observations indicate that the MNP datasets contain may interact with partially different molecular targets while simultaneously converging on common biological processes putatively associated with cellular stress responses.

Differences were also evident between the individual polymers. PE-responsive genes were predominantly associated with inflammatory signaling, oxidative stress, and apoptosis-related pathways, whereas PET-derived datasets showed a relatively greater enrichment of genes involved in lipid metabolism and metabolic regulation (Table 1). In contrast, the PS-derived dataset contained the largest and most diverse collection of toxicogenomic interactions, encompassing genes related to inflammation, oxidative stress, immune regulation, apoptosis, and various signaling pathways. PVC exhibited a comparatively focused toxicogenomic profile, with genes predominantly linked to antioxidant defense and inflammatory signaling, particularly those involved in theKEAP1– NRF2 regulatory axis (Table 1). Despite these differences, several molecular features were consistently observed in the analyzed polymers. Proinflammatory cytokines, including *IL1B*, *IL6*, and *TNF*, were repeatedly identified within the CTD-derived interaction networks. Similarly, oxidative stress-related genes including *CAT*, *HMOX1*, and *NFE2L2* appeared in several datasets. In addition, genes involved in apoptosis and cell survival, such as *AKT1*, *BAX*, and *BCL2*, have been identified in several polymers. These overlapping molecular features were primarily related to inflammatory signaling, redox regulation, and cell survival.

A comparison of functional enrichment analyses also showed both shared and polymer-specific biological features. Although the PE and PVC datasets showed relatively higher involvement of inflammatory and oxidative stress-related pathways, PET was characterized by the enrichment of lipid metabolic pathways, and the PS dataset contained a broader spectrum of pathways related to signal transduction and cancer-associated signaling. Taken together, available toxicogenomic evidence supports the possibility that polymer-specific molecular responses coexist with a set of conserved biological processes common to multiple MNP types.

Chemical similarity analysis provided an additional comparative perspective on the molecular profiles of the selected polymers. For example, PE and PVC appeared within each other’s chemical similarity profiles and shared interacting genes, including *IL6*, *KEAP1*, *NFE2L2*, and *TNF*, which were also present in their inflammatory and redox-related profiles. Firemaster BP-6 (CAS No. 59536-65-1), a polybrominated biphenyl flame retardant, was appeared in the similarity profiles of both PE and PVC and featured common inflammatory and redox-related genes, including IL6, TNF, and KEAP1. In the PET dataset, 2,2′,4,6,6′-pentachlorobiphenyl, a polychlorinated biphenyl, was also identified, with OCLN and TJP1 as mutual interacting genes. These examples illustrate that the CTD-derived chemical similarity profiles include compounds from halogenated chemical classes that share subsets of molecular interactions with the selected polymers, although the extent and biological relevance of these similarities varied among the analyzed datasets (Datas S1–S4).

Similarly, disease inference analyses identified overlapping links with several neoplastic disease categories for all four selected polymers. This pattern shows that genes curated within the CTD-derived datasets overlap with molecular networks implicated in tumor biology. Consequently, comparative toxicogenomic analysis is a useful tool for identifying molecular processes that warrant further investigation in experimental and epidemiological studies of the health effects of MNP exposure.

The comparative results provide an integrated overview of the molecular signatures reported for different MNP polymers. Such comparative toxicogenomic evidence provides a useful foundation for organizing existing molecular knowledge and discussing the biological processes that might link MNP exposure to carcinogenesis in the following section.

## 5. Common Molecular Processes Linking MNPs and Carcinogenesis

Comparative analysis of the CTD-derived toxicogenomic datasets identified several genes that were recurrently detected in the four selected MNPs. Among these, *IL1B*, *IL6*, *TNF* (inflammatory signaling), *CAT*, *HMOX1*, and *NFE2L2* (oxidative stress regulation) were consistently observed in the majority of the polymer datasets, indicating that inflammation and redox imbalance are common biological responses reported in relation to MNP exposure (Table 2). In addition, genes involved in cell survival and metabolic regulation appeared in the polymer-specific analyses, indicating that different MNPs might influence carcinogenesis through partially overlapping yet complementary molecular processes. Based on these observations, the following sections summarize the principal biological processes through which chronic MNP exposure may contribute to the development of cancer.

### 5.1. Oxidative Stress and Redox Imbalance

Oxidative stress is one of the most consistently reported biological responses to MNP exposure and has been proposed as an important mechanism that potentially links chronic MNP exposure to carcinogenesis [16,43,44]. Comparative analysis of the CTD-derived datasets revealed the recurrent identification of oxidative stress-responsive genes, including *CAT*, *HMOX1*, and *NFE2L2*, in the major MNP polymers (Table 2). These genes participate in the KEAP1-NRF2-mediated antioxidant defense system, which plays a central role in maintaining intracellular redox homeostasis, suggesting that oxidative stress-related pathways constitute a common toxicogenomic signature associated with MNP exposure.

This toxicogenomic profile is broadly aligned with experimental evidence demonstrating that MNP exposure induces excessive ROS generation; oxidative damage to DNA, proteins, and membrane lipids; mitochondrial dysfunction; and the activation of diverse stress-response pathways. Although the activation of antioxidant defense mechanisms initially protects cells against oxidative injury, persistent or excessive oxidative stress may overwhelm these protective systems, leading to genomic instability, accumulation of DNA damage, and dysregulation of cellular signaling pathways. Collectively, these observations support oxidative stress as one of the principal biological processes linking chronic MNP exposure to molecular alterations relevant to carcinogenesis. Importantly, oxidative stress does not act in isolation but is intimately linked with inflammatory signaling and cell survival pathways, and these interactions may further influence cancer-related molecular processes during chronic MNP exposure.

### 5.2. Chronic Inflammation and Immune Dysregulation

Proinflammatory cytokines, including IL1B, IL6, and TNF, have been consistently identified in experimental studies as key mediators of the chronic inflammatory response induced by MNP exposure, indicating that inflammatory signaling is one of the major biological processes that plausibly links prolonged MNP exposure to carcinogenesis [16,40,43,96]. Consistent with these experimental observations, a comparative analysis of the CTD-derived datasets revealed the recurrent occurrence of IL1B, IL6, and TNF within the selected MNP polymers, supporting that inflammatory signaling constitutes a common toxicogenomic signature associated with MNP exposure (Table 2).

This toxicogenomic profile is concordant with experimental evidence demonstrating sustained cytokine production and activation of the NF-κB, JAK/STAT, and MAPK signaling pathways following MNP exposure. Persistent activation of these pathways promotes oxidative stress, extracellular matrix remodeling, angiogenesis, and altered immune surveillance, thereby creating a tissue microenvironment that may favor tumor development and progression. Chronic inflammation can also enhance the interactions between immune cells and damaged tissues, resulting in the sustained production of inflammatory mediators that further amplify cellular stress responses and promote a pro-tumorigenic microenvironment. These observations support the hypothesis that chronic inflammatory signaling is one of the major mechanisms through which prolonged MNP exposure may influence molecular processes relevant to carcinogenesis. Given the extensive crosstalk between inflammatory signaling, oxidative stress, and cell survival pathways, persistent inflammation may further amplify cellular stress responses and promote molecular alterations that could collectively favor tumor initiation and progression during chronic MNP exposure.

### 5.3. Dysregulation of Cell Survival and Cell-Cycle Control

Genes involved in cell survival, apoptosis, and DNA damage responses, including *AKT1*, *BCL2*, and *TP53*, are widely recognized as critical regulators of cellular adaptation to environmental stress and are frequently dysregulated during tumor development [40]. These signaling molecules coordinately regulate the balance between cell survival, programmed cell death, and DNA damage responses, thereby preserving genomic integrity and cellular homeostasis under physiological conditions. The disruption of these regulatory networks has been implicated in the initiation and progression of a broad spectrum of human cancers.

Consistent with these experimental observations, comparative analysis of the CTD-derived datasets suggested the presence of AKT1, BCL2, and TP53, although their distribution varied depending on the polymer type (Table 2). These findings imply that the molecular pathways governing cell survival, apoptosis, and DNA damage responses may constitute another common toxicogenomic feature following MNP exposure. This toxicogenomic profile is consistent with experimental studies demonstrating that MNP exposure influences PI3K/AKT signaling, p53-mediated stress responses, and apoptosis-related pathways. Persistent dysregulation of these signaling networks may impair cell-cycle checkpoint control, reduce the elimination of damaged cells through apoptosis, and facilitate the accumulation of genomic alterations that favor malignant transformations. Although the current toxicogenomic evidence does not demonstrate a direct carcinogenic effect of MNP exposure, these observations support the hypothesis that prolonged MNP exposure may influence the molecular pathways that are commonly dysregulated during carcinogenesis.

Beyond alterations in cell survival and cell-cycle control, experimental studies have shown that MNP exposure, particularly exposure to nanoscale particles, can affect tumor-cell proliferation, migration, and invasion [56,57,97,98,99]. Enhanced proliferative and invasive phenotypes have been associated with alterations in signaling pathways involved in cell growth, survival, cytoskeletal remodeling, and extracellular matrix regulation [99,100,101]. These findings broaden the potential cancer-related implications of MNP exposure beyond cellular stress responses and suggest possible effects on cellular phenotypes relevant to tumor progression and metastatic behavior.

Importantly, the regulation of cell survival and cell cycle progression is closely associated with oxidative stress and inflammatory signaling. Persistent oxidative damage and chronic inflammatory responses may further disrupt these regulatory networks, indicating that these interconnected molecular processes may collectively contribute to genomic instability and create favorable biological conditions relevant to tumor initiation and progression during chronic MNP exposure.

### 5.4. Metabolic Reprogramming

Alterations in cellular metabolism, particularly lipid metabolism, are increasingly recognized as important features of adaptive responses to environmental stress and are closely associated with cancer development [40,102]. Metabolic reprogramming supports cellular survival by coordinating energy production, membrane biosynthesis, and redox homeostasis, and influences inflammatory and stress-response signaling pathways [103,104].

Based on these biological observations, a comparative analysis of the CTD-derived datasets suggested that the PET dataset showed the most prominent clustering of genes involved in lipid metabolism among the selected MNPs analyzed in this review (Table 2). The key genes included *APOA4*, *AGPAT1*, *GPAT4*, *LPCAT3*, *MOGAT3*, *MGLL*, *MFSD2A*, *HMGCS2*, and *PLCE1*, suggesting that metabolic regulation may define a relatively distinctive toxicogenomic feature of PET exposure (Table 3, Data S2). This toxicogenomic profile is consistent with experimental studies demonstrating that MNP exposure alters lipid metabolism, phospholipid remodeling, mitochondrial function, and cellular energy homeostasis. As metabolic pathways are tightly coupled with oxidative stress and inflammatory signaling, persistent metabolic dysregulation could further amplify cellular stress responses and promote adaptive processes that support cell survival under chronic exposure conditions. Although the current evidence remains limited, these observations support the possibility that metabolic reprogramming cooperates with oxidative stress, inflammatory signaling, and dysregulated cell survival to influence molecular processes relevant to carcinogenesis (Table 3).

### 5.5. Epigenetic Modificatins

Epigenetic alterations may represent an additional regulatory mechanism through which MNP exposure influences cellular responses relevant to carcinogenesis. Experimental studies have reported changed in MNP-associated changes in DNA methylation and non-coding RNA expression [99,105,106]. These regulatoru changes can influence gene involved in oxidative stress responses, inflammation, cell survival, and cell-cycle regulation.

Such modifications could provide a molecular link between persistent environmental exposure and sustained changes in cellular phenotype. In cancer biology, aberrant DNA methylation, histone modifications, and non-coding RNA regulation are closely associated with altered expression of tumor suppressor genes and oncogenic signaling pathways [107,108,109]. MNP-associated epigenetic changes could therefore intersect with the oxidative, inflammatory, metabolic, and cell-survival pathways described above. However, direct evidence establishing epigenetic alterations as a mechanism of MNP-associated carcinogenesis remains limited, and further studies under environmentally relevant chronic exposure conditions are required.

### 5.6. Combined Effects of Co-Existing Environmental Contaminants

Environmental MNPs can interact with co-existing contaminants through adsorption onto their surfaces, potentially altering the transport, bioavailability, and biological effects of both the particles and associated chemicals [110,111]. Such contaminants may include metals and diverse organic pollutants encountered in environmental matrices. Consequently, combined exposure to MNPs and co-existing contaminants may produce additive or potentially synergistic biological responses that differ from those induced by either component alone.

Several studies have reported enhanced oxidative stress, inflammatory responses, cellular damage, and genotoxic effects following co-exposure to MNPs and environmental contaminants [99,112,113,114]. The CTD phenotype datasets examined in this review also contained records involving MNPs together with different classes of environmental chemicals. These included PE and PVC microplastics co-treated with lead, PET-derived microplastics with perfluorooctanoic acid, and PS with diethylhexyl phthalate, with the corresponding phenotype annotations involving inflammatory responses, oxidative stress, intestinal barrier function, mitochondrial alterations, and metabolic processes (Datas S1–S4). Such combined exposures could intensify several of the cancer-relevant processes discussed above, including persistent oxidative damage, chronic inflammation, and disruption of cellular homeostasis. However, the magnitude and direction of such responses vary according to particle properties, contaminant type, exposure concentration, and biological model, emphasizing the importance of evaluating MNPs within environmentally relevant mixture-exposure scenarios.

### 5.7. Integrated Model of MNP Exposure and Carcinogenesis

**Figure 2:**
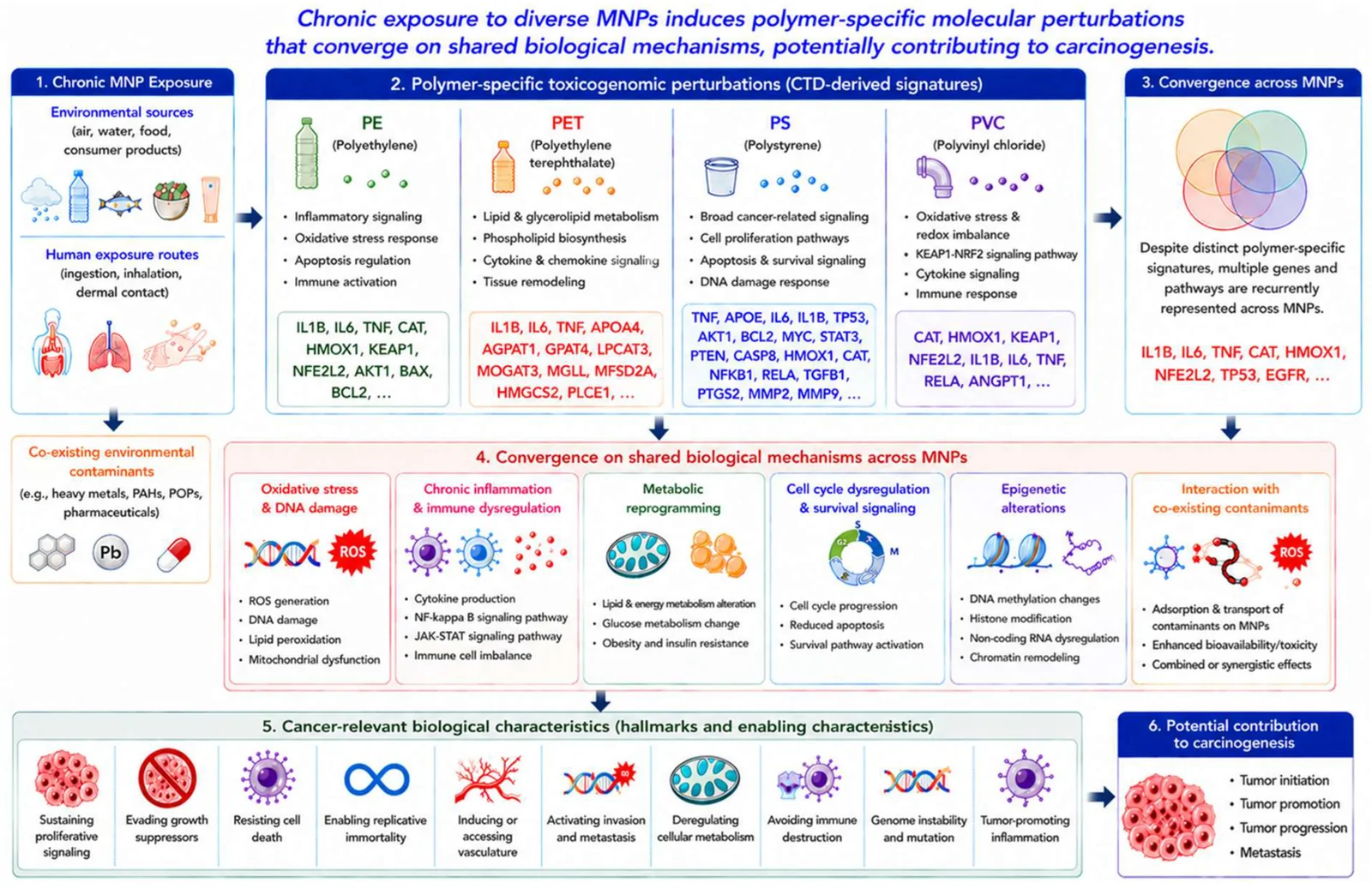
Proposed integrated model illustrating the potential molecular mechanisms linking chronic MNP exposure to carcinogenesis based on comparative CTD-derived toxicogenomic analysis. This schematic summarizes the conceptual model proposed in the present review based on comparative toxicogenomic analyses of representative MNP polymers using CTD. Chronic human exposure to environmentally relevant MNPs through ingestion, inhalation, and dermal contact may elicit polymer-specific molecular responses while simultaneously inducing shared molecular signatures observed in multiple polymer types. Co-existing environmental contaminants, including heavy metals, polycyclic aromatic hydrocarbons, persistent organic pollutants, and pharmaceuticals, are also presented as potential modifiers of MNP-associated biological effects. The upper panel illustrates the polymer-specific toxicogenomic characteristics identified for PE, PET, PS, and PVC. Despite distinct molecular signatures for each polymer, comparative CTD analyses identified several consistently shared genes, particularly inflammatory cytokines (IL1B, IL6, and TNF), oxidative stress-associated genes (CAT, HMOX1, and NFE2L2) together with other recurrent genes associated with cell survival and cancer-related signaling. The middle panel summarizes the principal biological processes emerging from these analyses, including oxidative stress and redox imbalance, chronic inflammation and immune dysregulation, dysregulation of cell survival and cell-cycle control, and metabolic reprogramming. These processes are functionally intertwined and may mutually reinforce one another through interconnected signaling networks. The lower panel illustrates how these molecular responses may contribute to cancer-relevant biological characteristics, including sustained proliferative signaling, resistance to cell death, genomic instability, tumor-promoting inflammation, angiogenesis, replicative immortality, and altered cellular energetics. Collectively, these processes provide a biologically plausible framework through which chronic MNP exposure could conceivably contribute to tumor initiation, promotion, and progression. Nevertheless, this model should be interpreted as a hypothesis-generating conceptual framework derived from curated toxicogenomic evidence rather than as definitive proof of a causal relationship between MNP exposure and carcinogenesis. CTD, Comparative Toxicogenomics Database; MNPs, micro- and nanoplastics; PE, polyethylene; PET, polyethylene terephthalate; PS, polystyrene; PVC, polyvinyl chloride; ROS, reactive oxygen species. Ellipses (…) indicate additional genes not displayed in the figure.

Based on available evidence, the comparative CTD analyses presented in this review suggest that polymer-specific toxicogenomic profiles share on a limited number of interconnected biological processes, including oxidative stress, chronic inflammation, dysregulated cell survival, metabolic reprogramming, and epigenetic modifications. Rather than functioning as independent mechanisms, these biological responses may interact through extensive molecular crosstalk and collectively influence cellular homeostasis during chronic MNP exposure. Interactions with co-existing environmental contaminants may further modify these molecular responses. Persistent activation of these cross-regulatory pathways may promote DNA damage, genomic instability, altered tissue homeostasis, immune dysregulation, and sustained cellular adaptation, thereby creating biological conditions that may favor tumor initiation and progression. Although the toxicogenomic signatures identified through CTD do not establish causal relationships, they provide a systems-level basis for understanding how diverse MNP polymers converge on common molecular processes in the context of carcinogenesis. Collectively, this comparative toxicogenomics perspective provides a systematic basis for prioritizing candidate molecular targets, signaling pathways, and biological processes for future mechanistic, experimental, and epidemiological investigations aimed at clarifying the long-term health consequences of chronic MNP exposure (Figure 2).

## 6. Pan-Cancer Analysis of CTD-Derived Candidate Genes

### 6.1. Differential Expression in Human Cancers

**Figure 3:**
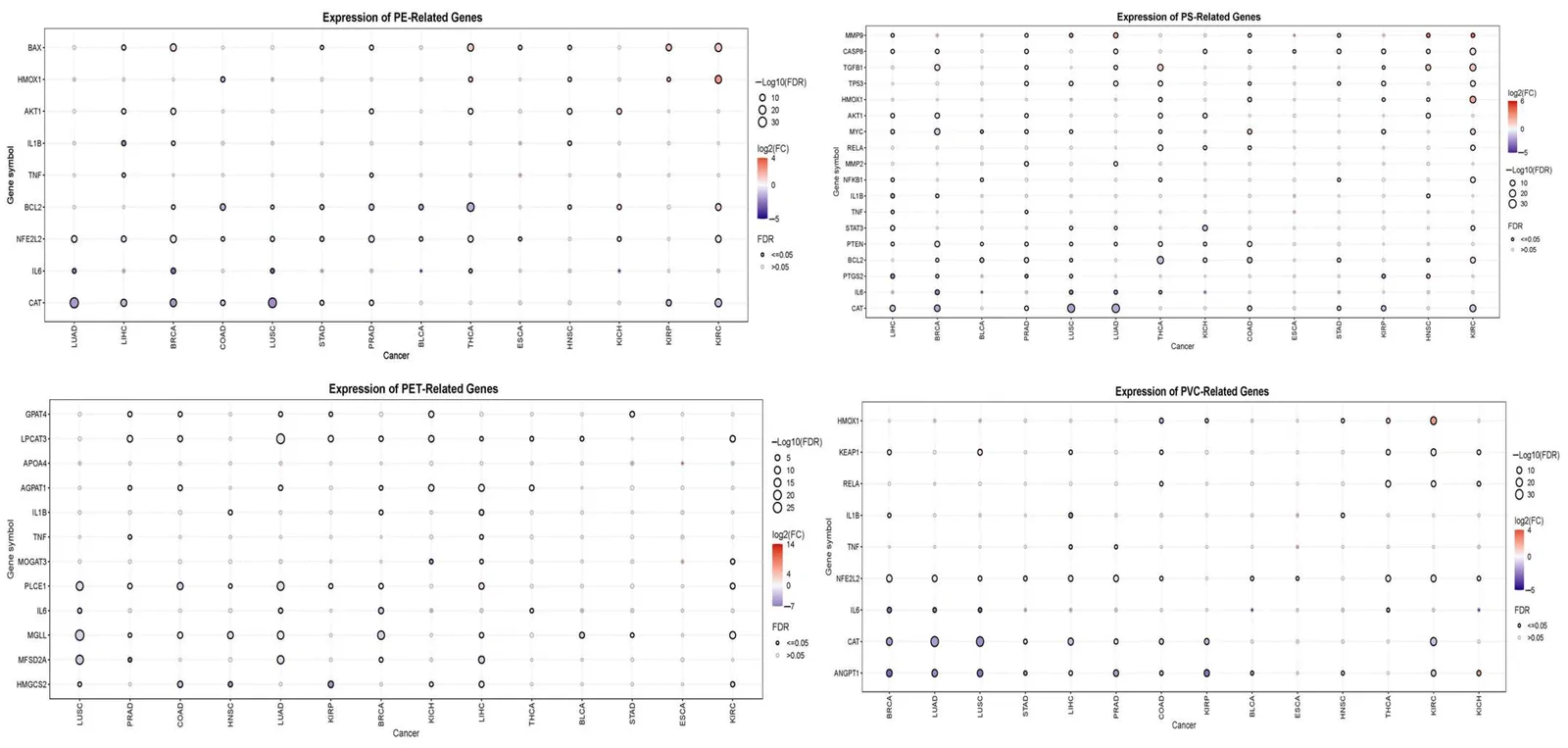
Pan-cancer differential expression of CTD-derived MNP-responsive genes. Differential expression of recurrent MNP-responsive genes was evaluated in tumor versus normal tissues using GSCA pan-cancer datasets. Bubble color indicates log_2_ fold change between tumor and normal tissues, whereas bubble size represents statistical significance (−log_10_ adjusted *p* value). Red bubbles, upregulated; Blue bubbles, downregulated.

Comparative toxicogenomic analysis identified several MNP-responsive genes that were consistently expressed in response to the selected polymers. To further evaluate their potential relevance in human cancers, Gene Set Cancer Analysis (GSCA; https://guolab.wchscu.cn/GSCA/#/; accessed on 16 July 2026) pan-cancer gene expression datasets were analyzed for representative CTD-derived candidate genes [94]. In several cancer types, numerous inflammatory and oxidative stress-related genes exhibit significant alterations in tumor tissues compared to corresponding normal tissues, suggesting that genes responsive to MNP exposure may also be frequently dysregulated in human malignancies. Of the recurrent inflammatory mediators, IL1B, IL6, and TNF demonstrated significant changes in expression in several tumor types, although the direction of dysregulation varied according to the cancer type (Figure 3, Table 4, and Data S5). For example, IL1B showed increased expression in head and neck squamous cell carcinoma (HNSC) but reduced expression in LIHC and breast cancer, suggesting that tissue-specific regulatory mechanisms may contribute to its expression. Similar context-dependent alterations were observed for IL6 and TNF in different malignancies. In parallel, oxidative stress-associated genes, including CAT, HMOX1, NFE2L2, and KEAP1, together with cell survival regulators, such as AKT1 and BCL2, also exhibited significant differential expression in various tumor types.

Collectively, these analyses suggest that several CTD-derived MNP-responsive genes also dysregulated in human cancers. Although these observations do not establish a causal relationship between MNP exposure and tumorigenesis, they may support the putative biological relevance of the candidate genes identified through comparative toxicogenomic analyses.

### 6.2. Association with Immune Infiltration

**Figure 4:**
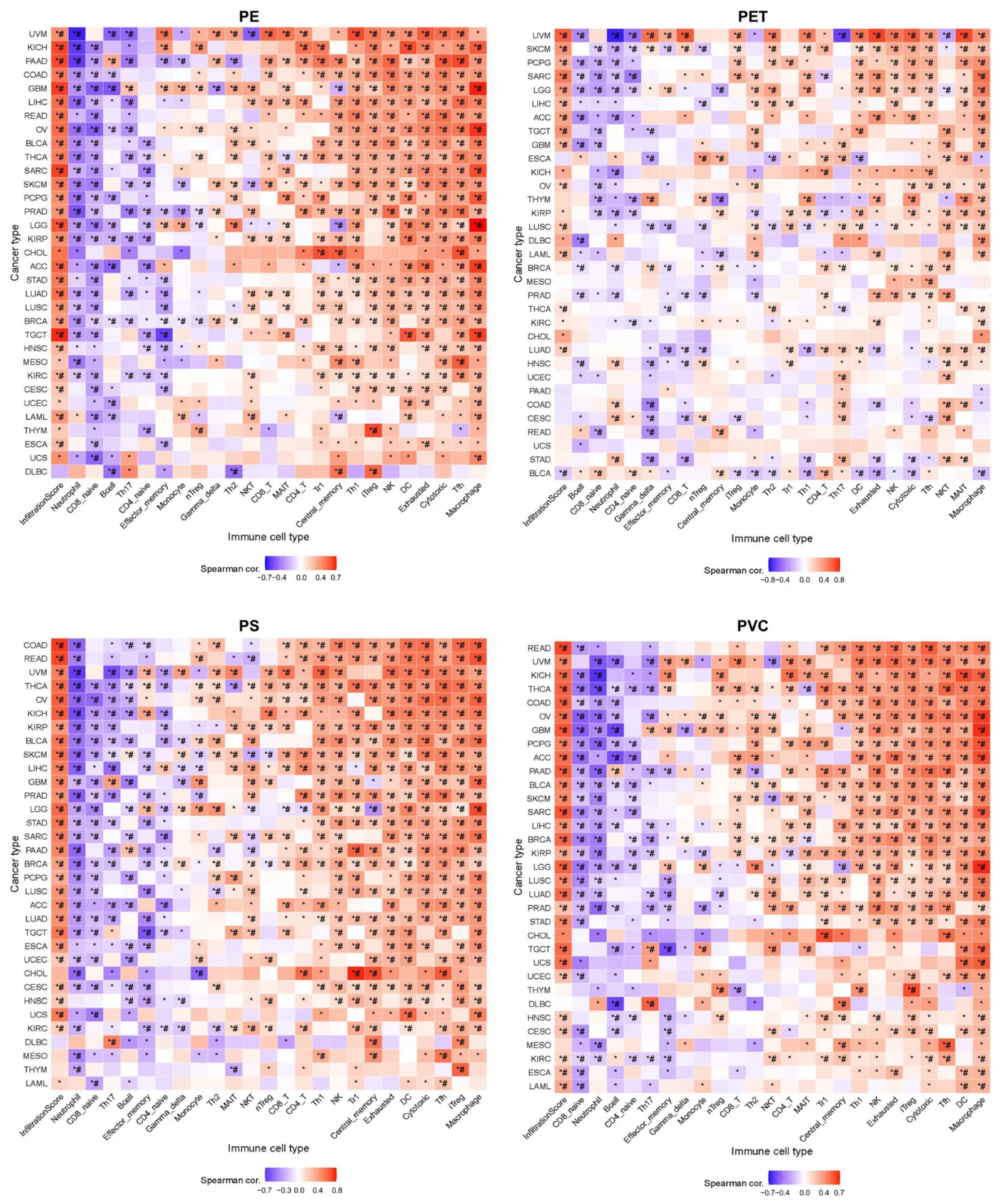
Pan-cancer immune cell infiltration correlations of CTD-derived MNP-responsive genes. Correlations between recurrent MNP-responsive genes and tumor-infiltrating immune cell populations were analyzed using GSCA pan-cancer datasets. Each panel represents the correlation pattern of an individual gene with immune cell subsets, including CD8+ cytotoxic T cells, CD4+ T cells, regulatory T cells (Tregs), memory T cells, NK cells, neutrophils, and macrophages. Colors represent Spearman correlation coefficients, with red denoting positive correlations and blue denoting negative correlations. Numerical values indicate correlation coefficients, and an asterisk denotes statistical significance (*p* < 0.05), and a hash symbol (#) denotes FDR < 0.05.

Because chronic inflammation and immune dysregulation emerged as common toxicogenomic signatures in multiple MNP polymers, the relationships between CTD-derived candidate genes and tumor immune infiltration were further explored using GSCA pan-cancer datasets. Overall, significant correlations were observed between recurrent MNP-responsive genes and numerous immune cell populations in diverse cancer types, suggesting that their expression is linked to distinct immune cell infiltration patterns within the tumor immune microenvironment. Inflammation-related genes, including IL1B, IL6, and TNF, displayed broad associations with both innate and adaptive immune cell populations, whereas oxidative stress regulators and survival-related genes also demonstrated significant relationships with cytotoxic T cells, NK cells, neutrophils, macrophages, regulatory T cells, and memory T cell subsets in a cancer type–dependent manner (Figure 4, Table 5, and Data S6). Notably, the direction and magnitude of these correlations varied considerably between individual tumors, reflecting the marked heterogeneity of the immune landscapes in different malignancies.

These observations are consistent with the CTD-derived toxicogenomic analysis, in which inflammatory signaling was persistently represented in the MNP-associated datasets. From a biological perspective, sustained alterations in inflammatory mediators such as IL1B, IL6, and TNF could theoretically modify the immune context of tumors. Changes in these cytokine-related profiles may affect macrophage recruitment and functional polarization and could also alter cytotoxic T-cell activity, potentially shifting the balance between antitumor and tumor-supportive immune responses. However, the GSCA datasets analyzed here contain no information on individual MNP exposure. Therefore, these correlations do not demonstrate that MNPs directly modify immune infiltration but rather provide a clinical context in which MNP-responsive molecular profiles intersect with immune characteristics of different tumor types.

### 6.3. Prognostic Significance in Cancers

**Figure 5:**
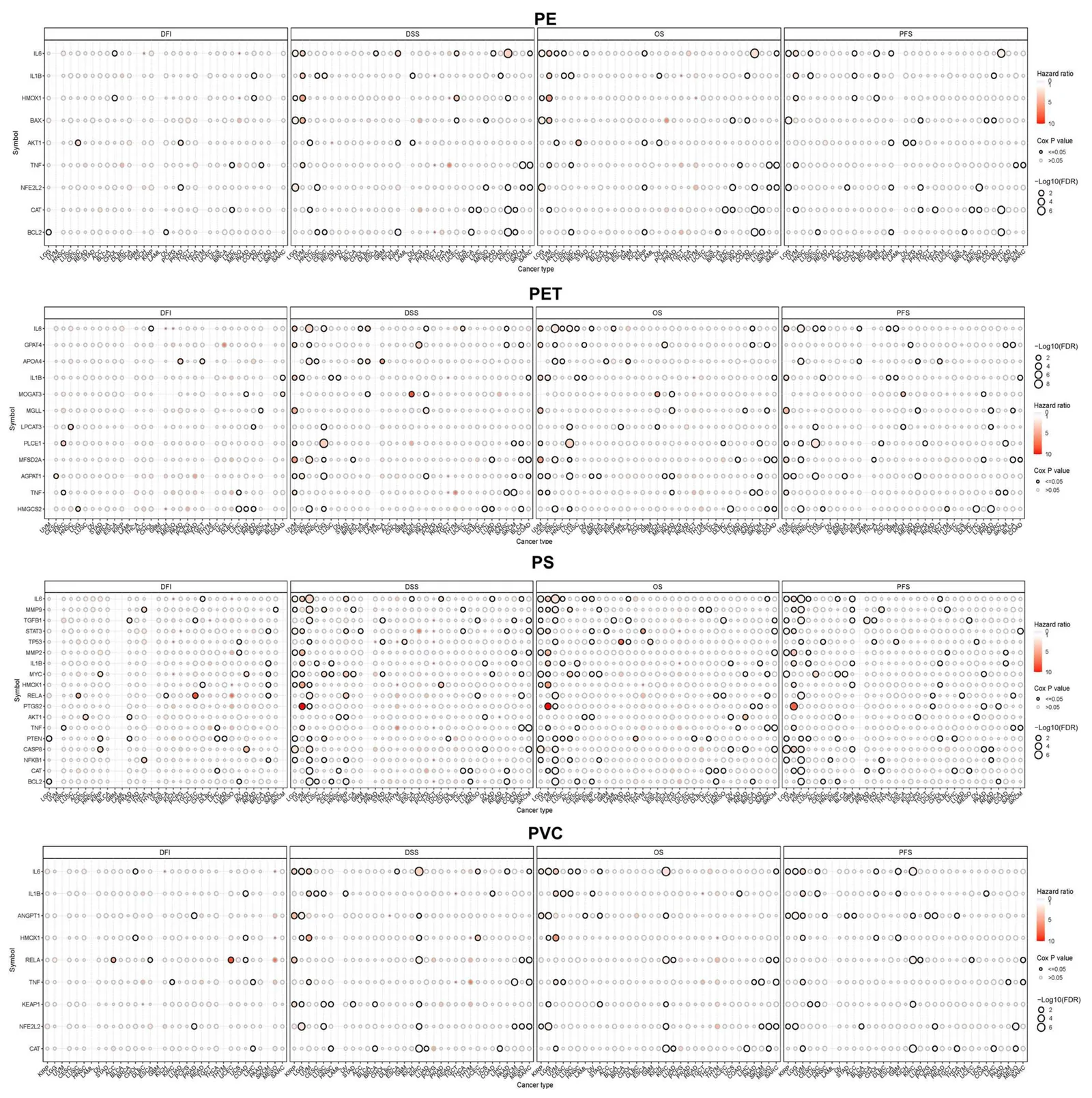
Pan-cancer survival analysis of CTD-derived MNP-responsive genes. Prognostic significance of recurrent MNP-responsive genes was estimated in TCGA cancer types using GSCA pan-cancer survival datasets. Bubble color indicates hazard ratio, and bubble size represents statistical significance (−log_10_ *p* value). OS, overall survival; DSS, disease-specific survival; PFS, progression-free survival; DFI, disease-free interval.

Survival analyses were performed for several human cancers using GSCA datasets to investigate the potential clinical significance of repeatedly observed CTD-derived candidate genes. Several recurrent MNP-responsive genes are significantly associated with patient prognosis in various cancer types, indicating that these genes may have prognostic relevance beyond their toxicogenomic responsiveness. Of the analyzed genes, IL1B, IL6, and TNF showed the broadest prognostic associations (Figure 5, Table 6, and Data S7). IL1B demonstrated a significant relationship with overall survival, progression-free survival, disease-specific survival, and disease-free interval in colorectal cancer, whereas consistent prognostic significance at numerous survival endpoints was observed in lung squamous cell carcinoma and uveal melanoma. Similarly, IL6 exhibited widespread prognostic associations in kidney clear cell carcinoma, kidney papillary cell carcinoma, lower-grade glioma, and uveal melanoma. TNF was likewise associated with numerous survival endpoints in sarcoma, skin cutaneous melanoma, and uveal melanoma. In addition, oxidative stress-related genes, such as CAT, showed significant associations with survival in several cancer types, including liver hepatocellular carcinoma, kidney renal clear cell carcinoma, and lung adenocarcinoma.

Overall, these findings indicate that several recurrent MNP-responsive genes identified through CTD-based toxicogenomic analyses have prognostic significance in diverse human malignancies. Although these clinical associations should not be interpreted as evidence that MNP exposure directly influences cancer prognosis, they provide additonal biological and clinical context for the molecular signatures identified in different MNP polymer datasets.

## 7. Limitations and Future Perspectives

The comparative toxicogenomic approach employed in this review provides a systematic integration of molecular evidence derived from selected MNPs; however, several limitations should be considered when interpreting the present findings. First, the toxicogenomic information available in CTD is derived from heterogeneous experimental studies that differ substantially in polymer type, particle size, surface characteristics, exposure concentration, exposure duration, biological model, and analytical methodology. Consequently, the CTD-derived toxicogenomic profiles summarized in this review should not be interpreted as direct comparisons under equivalent experimental conditions but rather as an integrated representation of currently available molecular evidence [47]. Second, CTD primarily provides curated chemical–gene interactions together with inferred disease and pathway relationships, rather than direct evidence of causal relationships [47,48]. Although recurrently identified genes and enriched biological pathways may reflect molecular responses reported following MNP exposure, these observations do not demonstrate that individual MNP polymers directly induce carcinogenesis. Similarly, although experimental studies have reported cancer-related biological effects of MPs and NPs, including alterations in oxidative stress, inflammatory responses, and tumor-associated cellular processes, the available evidence primarily supports potential associations and biological plausibility rather than a causal relationship with human cancer. Conclusive evidence that chronic MP or NP exposure directly contributes to cancer development or progression in humans is currently lacking. Therefore, the toxicogenomic signatures identified in the present analysis should be regarded as hypothesis-generating and require validation through mechanistic in vitro and in vivo studies, as well as well-designed human epidemiological investigations.

Notably, the pan-cancer analyses performed in this review were based on publicly available transcriptomic datasets rather than on MNP exposure-specific human cohorts, which is an inherent limitation of the present pan-cancer analysis. Therefore, the observed associations between candidate gene expression, immune cell infiltration, and patient prognosis do not provide direct evidence that MNP exposure influences these clinical characteristics. Nevertheless, the fact that the molecular signatures repeatedly identified in representative MNP polymers are also broadly represented in human cancers lends additional support to their potential biological relevance, even though these observations remain hypothetical with respect to MNP-associated carcinogenesis. Another important limitation is the unequal availability of toxicogenomic information for different MNP polymers. PS has been investigated considerably more extensively than the other polymers included in this review, resulting in a larger number of curated molecular interactions within CTD. In contrast, PP, despite being one of the most abundant environmental plastics, was not included in the comparative analysis because of the limited availability of PP-specific toxicogenomic information in the current CTD database. As additional experimental evidence becomes available, future CTD updates are expected to improve the completeness and balance of the comparative toxicogenomic analyses. An additional concern is that some experimental studies, particularly acute-exposure studies, have employed MNP concentrations substantially higher than those expected under realistic human exposure conditions [115,116]. Therefore, findings obtained at such high exposure levels should be interpreted cautiously when extrapolating them to human health. Furthermore, environmental MNP exposure rarely occurs as exposure to a single pristine polymer. Humans are chronically exposed to complex mixtures of weathered particles that differ in polymer composition, particle size, surface chemistry, aging status, and co-occurring environmental contaminants [52,117]. Therefore, future toxicogenomic investigations should incorporate environmentally relevant exposure scenarios, including aged MNPs, mixed-polymer exposures, co-exposure to environmental contaminants, and long-term low-dose exposure models that more closely reflect real-world conditions [118]. Studies integrating exposure assessments, multi-omics approaches, and clinical datasets from human populations will be particularly valuable for determining whether consistently identified MNP-responsive molecular signatures observed through comparative CTD analyses are reproducibly associated with cancer development and clinical outcomes.

Despite these limitations, the comparative CTD-based approach presented in this review provides a useful strategy for systematically organizing the heterogeneous molecular evidence derived from selected MNP polymers. Rather than establishing causal mechanisms, this approach facilitates the identification of recurrent molecular responses, prioritization of candidate genes and biological pathways, and the generation of biologically plausible hypotheses for future investigation. Further integration of CTD-derived toxicogenomic information with multiomics approaches, including transcriptomics, proteomics, metabolomics, single-cell sequencing, spatial transcriptomics, and human biomonitoring studies, is expected to contribute to a more comprehensive understanding of the molecular mechanisms by which chronic MNP exposure may influence human health and carcinogenesis [119,120].

## 8. Conclusions

MNPs are ubiquitous environmental contaminants that are increasingly detected in environmental matrices, food products, and human biological tissues. Although growing experimental evidence shows that MNP exposure induces diverse molecular and cellular responses, the substantial heterogeneity arising from polymer types, physicochemical properties, and experimental conditions complicates efforts to identify common molecular mechanisms that are potentially relevant to adverse health outcomes. In this review, CTD-derived toxicogenomic information was comparatively integrated for four selected environmental MNP polymers: PE, PET, PS, and PVC. Comparative analyses of chemical–gene interactions, disease relationships, GO enrichment, pathway enrichment, phenotype annotations, and chemical similarity profiles demonstrated polymer-specific toxicogenomic characteristics while simultaneously revealing several common molecular responses in the analyzed datasets.

Among several commonly observed genes identified in the four polymers, *IL1B*, *IL6*, *TNF*, *CAT*, *HMOX1*, and *NFE2L2* consistently emerged as common molecular signatures. Polymer-specific characteristics, including genes involved in lipid metabolism, oxidative stress responses, inflammatory signaling, and cell survival pathways, further indicated that different MNP polymers were linked to partially distinct biological processes and common molecular responses involving oxidative stress, chronic inflammation, dysregulated cell survival, and metabolic remodeling. Subsequent pan-cancer analyses demonstrated that many of these candidate genes were broadly dysregulated in diverse human malignancies and exhibited significant associations with tumor immune infiltration and patient prognosis. These observations do not establish that MNP exposure directly contributes to cancer development or clinical outcomes but provide additional support for the biological and potential clinical relevance of the shared molecular signatures identified through comparative toxicogenomic analyses.

Importantly, the toxicogenomic evidence summarized in this review should be interpreted as hypothesis-generating rather than as definitive evidence of carcinogenicity. Nevertheless, the comparative evaluation of convergent CTD-derived molecular signatures provides a systematic basis for identifying biological processes and candidate molecular targets that warrant further mechanistic investigation. Rather than proposing a direct causal relationship between MNP exposure and carcinogenesis, this review presents a comparative CTD-based toxicogenomic perspective to systematically identify the recurrent molecular responses shared by selected MNP polymers. Collectively, this review provides a rational basis for prioritizing candidate genes and biological pathways for future mechanistic studies and may contribute to improving the molecular understanding of chronic MNP exposure, as well as future human health risk assessment.

## Figures and Tables

**Table 1:** Comparative toxicogenomic characteristics of selected MNP polymers.

Polymer	Featured * Genes	Major GO Biological Processes	Major Pathways	Representative CTD-Associated Diseases	Major Toxicogenomic Characteristics
PE	*IL1B*, *IL6*, *TNF*, *CAT*, *HMOX1*, *KEAP1*, *NFE2L2*, *AKT1*, *BAX*, *BCL2*	Negative regulation of apoptotic process; Negative regulation of programmed cell death; System development	Cytokine signaling; Signaling by Interleukins; Immune System; Endocrine resistance	Breast neoplasms; Prostatic neoplasms; Urinary bladder neoplasms; Neoplasm invasiveness; Neoplastic cell transformation	Inflammatory signaling, oxidative stress, and apoptosis-related responses
PET	*IL1B*, *IL6*, *TNF*, *APOA4*, *AGPAT1*, *GPAT4*, *LPCAT3*, *MOGAT3*, *MGLL*, *MFSD2A*, *HMGCS2*, *PLCE1*	Glycerolipid metabolic process; Cellular lipid metabolic process; Lipid metabolic process	Metabolic pathways; Lipid and lipoprotein metabolism; Phosphatidylinositol signaling; Inositol phosphate metabolism	Breast neoplasms; Lung neoplasms; Stomach neoplasms; Hepatocellular carcinoma	Lipid metabolism together with inflammatory signaling
PS	*TNF*, *APOE*, *IL6*, *IL1B*, *TP53*, *AKT1*, *BCL2*, *MYC*, *STAT3*, *PTEN*, *CASP8*, *HMOX1*, *CAT*, *NFKB1*, *RELA*, *TGFB1*, *PTGS2*, *MMP2*, *MMP9*	Cellular process; Response to stimulus; Metabolic process; Biological regulation	Pathways in Cancer; Signal Transduction; Immune System; Signaling by Interleukins; TNF signaling pathway	Obesity; Hypertension, Inflammation; Non-alcoholic fatty liver disease; Chemical- and drug-induced liver injury; Kidney diseases	Inflammatory, oxidative stress, apoptosis, and cancer-related signaling
PVC	*CAT*, *HMOX1*, *KEAP1*, *NFE2L2*, *IL1B*, *IL6*, *TNF*, *RELA*, *ANGPT1*	Negative regulation of apoptotic process; Negative regulation of cell death; Tube development; Angiogenesis; Response to hydrogen peroxide	Signaling by Interleukins; Immune System; IL-17 signaling; Fluid shear stress and atherosclerosis	Lung neoplasms; Hepatocellular carcinoma; Liver neoplasms; General neoplasms	Oxidative stress and inflammatory signaling

* indicates the major CTD-derived responsive genes discussed in the present review and is not intended to represent the complete ranked gene lists available in CTD.

**Table 2:** Cross-polymer genes consistently identified in selected MNP polymers.

Gene	Representative Pathway	Potential Biological Relevance	Detected in
*IL1B*	NF-κB	Inflammation	PE, PET, PS, PVC
*IL6*	JAK/STAT	Inflammation	PE, PET, PS, PVC
*TNF*	TNF signaling	Inflammation	PE, PET, PS, PVC
*CAT*	ROS detoxification	Oxidative stress	PE, PS, PVC
*HMOX1*	KEAP1–NRF2	Redox homeostasis	PE, PS, PVC
*NFE2L2*	KEAP1–NRF2	Antioxidant defense	PE, PS, PVC
*AKT1*	PI3K/AKT	Cell survival	PE, PS
*TP53*	DNA damage response	Genome stability	PS
*BCL2*	Apoptosis	Cell survival	PE, PS

**Table 3:** Polymer-specific versus shared biological mechanisms.

Mechanism	PE	PET	PS	PVC	Featured Genes
Oxidative stress	+++	+	+++	+++	*CAT*, *HMOX1*, *NFE2L2*
Inflammation	+++	++	+++	+++	*IL1B*, *IL6*, *TNF*
Cell survival	++	+	+++	+	*AKT1*, *BCL2*, *TP53*
Lipid metabolism	-	+++	+	-	*APOA4*, *AGPAT1*
Immune regulation	++	+	+++	++	*TNF*, *RELA*

+++ = highly enriched, ++ = moderately enriched, + = weakly enriched, - = not enriched. Footnote: Relative enrichment is based on a comparative interpretation of CTD-derived chemical–gene interactions, GO enrichment, pathway enrichment, disease inference, and phenotype annotations and does not represent the quantitative effect size.

**Table 4:** Summary of pan-cancer differential expression of CTD-derived MNP-responsive genes.

Gene	Associated Polymer(s)	Major Toxicogenomic Feature	Pan-Cancer Expression Pattern	Significant Cancer Types
*IL1B*	PE, PET, PS, PVC	Inflammatory signaling	Frequently dysregulated	BRCA, LIHC, HNSC, COAD
*IL6*	PE, PET, PS, PVC	Inflammatory signaling	Frequently dysregulated	LIHC, LUAD, BRCA
*TNF*	PE, PET, PS, PVC	Inflammatory signaling	Differential expression	HNSC, PRAD, LIHC
*CAT*	PE, PS, PVC	Oxidative stress	Altered expression	LIHC, KIRC, LUAD
*HMOX1*	PE, PS, PVC	Redox homeostasis	Frequently upregulated	LIHC, PAAD
*NFE2L2*	PE, PS, PVC	Antioxidant defense	Dysregulated	LUSC, LIHC
*AKT1*	PE, PS	Cell survival	Altered	BRCA, BLCA
*BCL2*	PE, PS	Anti-apoptosis	Dysregulated	BRCA, LUSC
*TP53*	PS	DNA damage response	Frequently altered	Multiple cancers
*APOA4*	PET	Lipid metabolism	Reduced in several cancers	LIHC, COAD
*AGPAT1*	PET	Lipid metabolism	Differential	LIHC
*ANGPT1*	PVC	Angiogenesis	Predominantly decreased	BLCA, BRCA

Abbreviations: BLCA, bladder urothelial carcinoma; BRCA, breast cancer; COAD, colon adenocarcinoma; HNSC, head and neck squamous cell carcinoma; KIRC, kidney renal clear cell carcinoma; LIHC, liver hepatocellular carcinoma; LUAD, lung adenocarcinoma; LUSC, lung squamous cell carcinoma; PAAD, pancreatic adenocarcinoma; PRAD, prostate adenocarcinoma.

**Table 5:** Association of selected MNP-responsive genes with immune infiltration in cancers.

Gene	Associated Polymer(s)	Major Immune Cells	Correlation	Related Biological Function
*IL1B*	PE, PET, PS, PVC	Macrophages, Neutrophils, Tregs	Variable	Chronic inflammation
*IL6*	PE, PET, PS, PVC	CD8 T cells, Macrophages	Variable	Cytokine-mediated immunity
*TNF*	PE, PET, PS, PVC	NK cells, CD8 T cells	Variable	Immune regulation
*CAT*	PE, PS, PVC	CD8 T cells	Moderate	Oxidative stress–immune crosstalk
*HMOX1*	PE, PS, PVC	Macrophages	Positive	Redox-associated immunity
*NFE2L2*	PE, PS, PVC	NK cells	Mixed	Antioxidant response
*APOA4*	PET	B cells	Weak	Metabolic regulation
*AKT1*	PE, PS	Central memory T cells	Mixed	Cell survival–immune interaction
*ANGPT1*	PVC	Endothelial-associated immune cells	Mixed	Angiogenesis

**Table 6:** Prognostic significance of CTD-derived MNP-responsive genes in human cancers.

Gene	Associated Polymer(s)	Significant Cancers	Significant Endpoints	Potential Clinical Implication
*IL1B*	PE, PET, PS, PVC	COAD, LUSC, UVM	OS, DSS, PFS, DFI	Prognostic inflammatory biomarker
*IL6*	PE, PET, PS, PVC	KIRC, KIRP, LGG	OS, DSS, PFS	Cytokine-associated prognosis
*TNF*	PE, PET, PS, PVC	SARC, SKCM, UVM	OS, DSS, PFS	Immune prognostic marker
*CAT*	PE, PS, PVC	LIHC, LUAD, KIRC	OS	Oxidative stress-associated prognosis
*HMOX1*	PE, PS, PVC	Multiple cancers	OS/PFS	Redox-associated prognosis
*AKT1*	PE, PS	Multiple cancers	OS	Cell survival
*TP53*	PS	Multiple cancers	OS	Genome instability
*APOA4*	PET	LIHC	OS	Lipid metabolism-related prognosis
*ANGPT1*	PVC	BLCA	OS	Angiogenesis-associated prognosis

Abbreviations: BLCA, bladder urothelial carcinoma; COAD, colon adenocarcinoma; KIRC, kidney renal clear cell carcinoma; KIRP, kidney renal papillary cell carcinoma; LIHC, liver hepatocellular carcinoma; LGG, brain lower-grade glioma; LUAD, lung adenocarcinoma; LUSC, lung squamous cell carcinoma; SARC, sarcoma; SKCM, skin cutaneous melanoma; OS, overall survival; DSS, disease-specific survival; PFS, progression-free survival; DFI, disease-free interval.

## Data Availability

All data are available on reasonable request to the corresponding author.

## Supplementary Materials

The following supporting information can be downloaded at: https://www.mdpi.com/article/10.3390/ijms27188223/s1.

## Abbreviations

The following abbreviations are used in this manuscript:

CTD	Comparative Toxicogenomics Database
GO	Gene Ontology
GSCA	Gene Set Cancer Analysis
MPs	microplastics
MNPs	micro- and nanoplastics
NPs	nanoplastics
PE	polyethylene
PET	polyethylene terephthalate
PP	polypropylene
PS	polystyrene
PVC	polyvinyl chloride
ROS	reactive oxygen species
